# Examination of Web-Based Single-Session Growth Mindset Interventions for Reducing Adolescent Anxiety: Study Protocol of a 3-Arm Cluster Randomized Controlled Trial

**DOI:** 10.2196/41758

**Published:** 2023-03-17

**Authors:** Shimin Zhu, Samson Tse, Ko Ling Chan, Paul Lee, Qijin Cheng, Jessica Sun

**Affiliations:** 1 Department of Applied Social Sciences The Hong Kong Polytechnic University Hong Kong Hong Kong; 2 Department of Social Work and Social Administration The University of Hong Kong Hong Kong Hong Kong; 3 Southampton Clinical Trials Unit University of Southampton Southampton United Kingdom; 4 Department of Social Work Hong Kong Hong Kong; 5 Castle Peak Hospital Hong Kong Hong Kong

**Keywords:** growth mindset, fixed mindset, mental health, secondary school students, brief intervention, belief-in-change

## Abstract

**Background:**

Anxiety disorders are the most common mental disorders worldwide. In Hong Kong, 7% of adolescents are diagnosed with anxiety disorders, and 1 in every 4 secondary school students reports clinical-level anxiety symptoms. However, 65% of them do not access services. Long waitlists in public services, the high cost of private services, or the fear of being stigmatized can hinder service access. The high prevalence of anxiety and low intervention uptake indicate a pressing need to develop timely, scalable, and potent interventions suitable for adolescents. Single-session interventions (SSIs) have the potential to be scalable interventions for diagnosable or subclinical psychopathology in adolescents. Providing precise and context-adapted intervention is the key to achieving intervention efficacy.

**Objective:**

This study aims to compare the effectiveness of three SSIs: single-session intervention of growth mindset on negative emotions (SIGMA), SSI of growth mindset of personality (SSI-GP), and active control, in reducing adolescent anxiety.

**Methods:**

Adolescents (N=549, ages 12-16 years) from secondary schools will be randomized to 1 of 3 intervention conditions: the SIGMA, SSI-GP, or active control. The implementation of each intervention is approximately 45 minutes in length. Adolescent participants will report anxiety symptoms (primary outcome), perceived control, hopelessness, attitude toward help-seeking, and psychological well-being at preintervention, the 2-week follow-up, and the 8-week follow-up. A pilot test has confirmed the feasibility and acceptability of SIGMA among adolescents. We hypothesized that SIGMA and SSI-GP will result in a larger reduction in anxiety symptoms than the control intervention during the posttest and 8-week follow-up period. We also predict that SIGMA will have a more significant effect than SSI-GP. We will use the intention-to-treat principle and linear regression-based maximum likelihood multilevel models for data analysis.

**Results:**

This study will be conducted from December 2022 to December 2023, with results expected to be available in January 2024.

**Conclusions:**

This protocol introduces the implementation content and strategies of growth mindset SSIs (consists of 2 forms: SIGMA and SSI-GP) among school students. The study will provide evidence on the efficacy of different growth mindset SSIs for adolescent anxiety. It will also establish implementation strategies for self-administrative SSIs among school students, which can serve as a pioneer implementation of a scalable and self-accessible brief intervention to improve the well-being of young people.

**Trial Registration:**

ClinicalTrials.gov NCT05027880; https://clinicaltrials.gov/ct2/show/NCT05027880

**International Registered Report Identifier (IRRID):**

PRR1-10.2196/41758

## Introduction

### Background

Anxiety is one of the leading causes of illness and disability among adolescents aged 10-19 years [1]. In the past decade, Hong Kong recorded a 6.9% prevalence rate of anxiety disorders with severe impairment [2], and 25% of youths were experiencing high subclinical anxiety symptoms in a 3-month period and required a clinical intervention [3]. Based on the prevalence and local youth population data [4], it can be roughly estimated that in Hong Kong there are 85,000 secondary students requiring help and intervention for their anxiety symptoms.

However, an estimated 65% of individuals with general anxiety disorders in Hong Kong do not access mental health services [5]. The median waiting time between the onset of symptoms and receiving public child and adolescent psychiatric service is 58 weeks, and the cost of seeking private treatment is reported HK$3000 (US $385) per monthly consultation, which means that many families have no choice but to wait for public services [6]. Lengthy waitlists or costly clinic-based treatments provided by highly trained mental health professionals may be difficult to disseminate on a broad scale. This traditional setting makes access to service more difficult in special circumstances, such as during the COVID-19 pandemic [7]. Moreover, adolescents with mental health symptoms are particularly vulnerable to stigma and discrimination and may not actively seek school-based interventions [8]. Even among youth who do access care, most drop out prematurely, completing just over 3 therapy sessions on average [9]. There is clearly a need to develop briefer, scalable, nonstigmatizing, and youth-friendly interventions for adolescents with general anxiety symptoms.

Single-session interventions (SSIs) are promising to be scalable, accessible, and cost-effective for youth’s mental health needs. SSIs refer to structured programs that intentionally involve only 1 visit or encounter with a clinic, provider, or program [10]. SSIs may serve as stand-alone or adjunctive clinical services. Emerging evidence supports the efficacy of SSIs in reducing and preventing youth’s mental health symptoms. For example, Schleider and Weisz [11] found that growth mindset SSIs led to more significant improvement in youth depression and perceived behavioral control in a 9-month follow-up than in active control. They also found that enhancing belief-in-change of personality-enhanced treatment access for adolescent depression [12], but recorded nonsignificant changes in general anxiety, social anxiety, and conduct problems [13].

A brief intervention can be effective if the intervention precisely addresses the underlying psychological process that contributes to the social or psychological problems [14]. Walton [14] and Wilson [15] underscore the importance of precise intervention and adaptive context to secure the potency of brief interventions. Brief interventions targeted to remove specific psychological barriers and produce recursive dynamics will affect long-term outcomes. Thus, the effectiveness of SSI is highly related to the implementation content (what to intervene) and strategies (how to implement). However, the existing SSIs vary in the effect size of effectiveness. Examining psychological mechanisms that address mental health more precisely and designing and implementing the intervention carefully will improve the efficacy of growth mindset SSIs.

This proposal aims to develop and examine the efficacy and effectiveness of growth mindset SSIs for adolescent anxiety in the Chinese context. It will advance the extant literature by implementing and comparing different domains of growth mindset and developing implementation strategies for SSI among Chinese adolescents. The existing growth mindset SSIs teach adolescents the growth mindset of personality (SSI-GP), which means the person can change. However, the effectiveness of SSIs for reducing anxiety was not as significant as reducing depression. Recently, Zhu et al [16,17] found the fixed mindset of negative emotions closely associated with adolescent depression and anxiety. Therefore, an intervention promoting growth mindsets of negative emotions may be a more precise factor in alleviating the worry and anxiety of adolescents, but it is not examined yet. Thus, this protocol presents our newly developed intervention: the single-session intervention for growth mindset on negative emotions (SIGMA) and compares its effectiveness with the SSI-GP and an active control.

**Figure 1:**
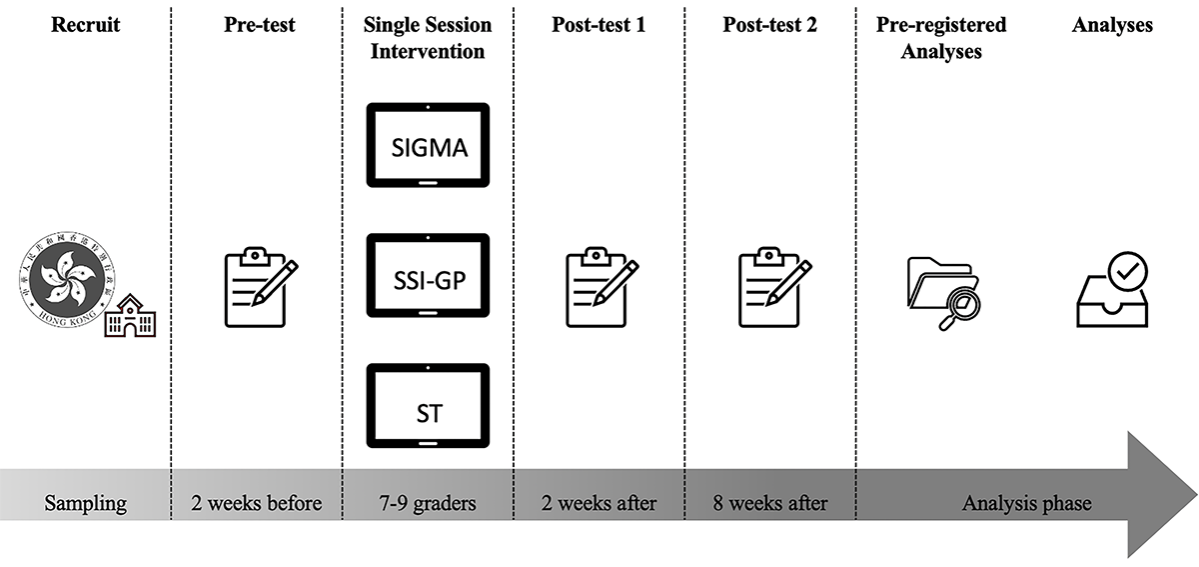
Design of the 3-arm randomized controlled trial. SIGMA: single-session intervention of growth mindset for anxiety; SSI-GP: single-session intervention of growth mindset of personality; ST: support therapy.

The SIGMA, which aims to instill the belief that negative emotions can change, may address the need for a scalable intervention for reducing anxiety. It has 3 unique features. First, it is a mechanism-targeted intervention with a carefully constructed and theoretically precise program that directly addresses the belief of suffering symptoms. Therefore, it may also advance the existing growth mindset SSI in reducing worry about symptoms and increasing perceived control and hope. Second, SIGMA can be developmentally adapted and culturally adapted for adolescents. It will be particularly suitable for secondary school students with general anxiety due to the engaging nature of web-based interventions, videos, peer sharing, and nonstigmatizing feature. Third, the brevity and flexible format could make it attractive to those who might not otherwise access care, and for whom a targeted, “light touch” intervention might be just enough. Given the large need-to-access gap among youths, we propose this 3-arm randomized controlled trial to provide evidence on the effectiveness of SIMGA and compare it against the existing growth mindset intervention (SSI-GP) and support theory as an active control. See Figure 1 for the research design.

### Aims and Objectives

The overarching goal of this study is to build the most effective implementation strategy of web-based growth mindset SSIs for reducing adolescent anxiety.

The primary objective of the proposed study is to evaluate the effectiveness of a SIGMA on reducing general anxiety symptoms in secondary school students. By using a 3-arm randomized control trial, we will compare the effectiveness of SIGMA to the SSI using growth mindset of personality (SSI-GP) and an active control condition using support therapy (ST).

The secondary objective is to compare the effectiveness of the aforementioned programs on secondary outcomes, including reducing hopelessness and increasing perceived control over emotions, attitude toward help-seeking, and psychological well-being.

There are 3 study hypotheses: (1) SIGMA and SSI-GP are more effective than the active control using ST in the primary outcomes, reducing general anxiety symptoms, and secondary outcomes, reducing hopelessness, enhancing perceived control, increasing positive attitude to help-seeking, and enhancing psychological well-being; (2) SIGMA is more effective than SSI-GP in the above primary and seconday outcomes; and (3) the effectiveness of SIGMA is greater in participants with higher motivation for change than in those with low to no motivation.

## Methods

### Research Design

**Figure 2:**
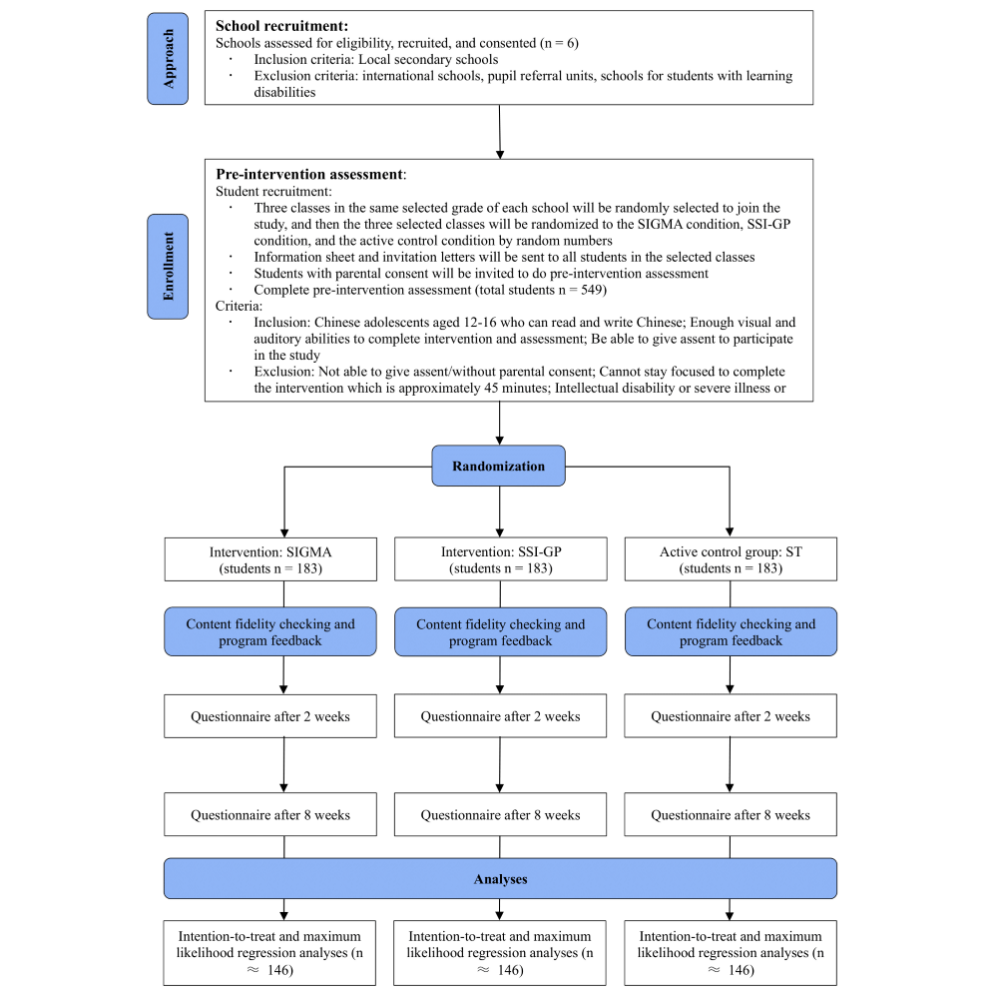
CONSORT (Consolidated Standards of Reporting Trials) diagram reflecting the flow of participants through this study. SIGMA: single-session intervention of growth mindset for anxiety; SSI-GP: single-session intervention of growth mindset of personality; ST: support therapy.

This study will use a cluster-randomized control trial design. A conceptual model is provided in Figure 1. The intervention protocol will be pre-registered before data collection in ClinicalTrials.gov (ref: NCT05027880), and strictly follow the CONSORT (Consolidated Standards of Reporting Trials) guidelines [18,19]. At least 3 classes in the same grade of the participating school will be randomized (using computer-generated random numbers) into the (1) SIGMA group, (2) SSI-GP group, and (3) ST group (active control condition group), which will provide ST intervention at the same time. All participants will receive regular interventions in school. Three repeated assessments of the measures will be conducted for the 3 groups simultaneously at (1) baseline, (2) 2-week postintervention, and (3) 8-week postintervention (Figure 2). The cluster randomization at the classroom level can balance the risk of contamination between the intervention and active control groups and the school heterogeneity due to the school culture, schedule, and management.

### Sample Size Determination

To make sure the sample size is big enough to test the hypotheses, a small to medium effect size (Cohen *d*=0.33) is used based on prior research [11], power set at 0.80, and α set at .05. A final sample size of 438 (146 per arm) is required. With reference to the attrition rate of our previous studies in a school setting, which is less than 20%, there is a requirement to recruit 549 participants at the baseline (183 per arm).

### Participant Eligibility and Randomized Control Process

Eligible participants (aged 12-16 years) in grades 7-9 will be recruited from 6 secondary schools through cluster randomized sampling. The inclusion criteria will include (1) ages between 12 and 16 years, (2) Chinese youth who can read and write Chinese, (3) enough visual and auditory abilities to complete the intervention and assessment, and (4) ability to give assent to participate in the study.

Exclusion criteria will include (1) no parental consent; (2) inability to stay focused to complete the intervention, which is approximately 45 minutes; and (3) intellectual disability or severe illness or pain that would lead to significant bias in students’ health and mental health situation. Eligible participants will not be screened for their anxiety symptoms; thus, this study will comprehensively examine the efficacy of the interventions for students with mild, moderate, and severe levels of anxiety.

The school and student recruitment process will include the following steps. First, we will send research invitations to schools chosen randomly from the school list. Invitations will stop when 6 schools agree to participate. At least 3 classes in the same selected grade of each school will be randomly selected to join the study, and then the 3 selected classes will be randomized to the SIGMA condition, SSI-GP condition, and the active control condition by random numbers. All students in those classes will be invited to participate and the final participation depends on parental consent and students’ assent. Based on prior trials in local schools, about 60% of students are likely to agree to participate with parental consent. Thus, approximately 90 students will be recruited from each school.

After consenting, students will be asked to complete a battery of questionnaires (detailed below) via the Qualtrics survey system. The students in 1 class will be in the same intervention conditions. The interventions will be conducted separately for each group in the school activity rooms with sufficient computers or tablets and headphones. All intervention activities are self-administered and delivered in a web-based format. The principal investigator and well-trained research assistants will stay in the intervention rooms to provide guidance and help if needed. All groups in 1 school will be conducted concurrently or within 2 weeks to reduce the influence of the time factor.

### Implementation Content

#### SIGMA

The SIGMA intervention group will adapt the SSI-GP protocol in two ways: (1) by introducing the growth mindset of emotions rather than personality and (2) by providing an experiential process of negative emotion change. SIGMA also consists of five components: (1) an introduction to emotions and the brain for conveying a scientific understanding of emotion and growth mindset of negative emotions; (2) stories and testimonials from high school–aged youths who described their beliefs that people’s negative emotion states (eg, anxiety, depression, and stress) are malleable, and how these mindsets influence their coping with anxiety; (3) emotion changing experience induced by short videos; (4) common questions and misconceptions about growth mindset; and (5) self-persuasion writing exercises in which the participants write notes to younger students about the growth mindset of emotion. Table 1 presents the key elements of the interventions. For participants at each school who belong to the SIGMA group, we will randomly select half of them to give them booster messages with core intervention content every 2 weeks between the 2-week posttest and the 2-month follow-up survey, that is, a total of 5 weekly booster messages will be sent to half of the participants of the intervention group, which will help us determine the most effective way of implementing the intervention.

**Table 1:** Intervention elements.

Intervention elements	SIGMA^a^ (45 minutes)	SSI-GP^b^ (45 minutes)	ST^c^ (45 minutes)
Science knowledge about neural plasticity of the brain	✓ Growth mindset of negative emotions	✓ Growth mindset of personality	Nil Science videos about emotions
Stories and testimonials from high school-aged youths	✓ Focused on emotion changing experience	✓ Focused on personality change	✓ Focused on emotion sharing
Experiential exercises	✓ Emotion coping strategies	Nil	Nil
Self-persuasion writing exercises	✓ Focused on the malleability of negative emotion	✓ Focused on the malleability of personality	✓ Focused on sharing emotion to close others

^a^SIGMA: single-session intervention of growth mindset on negative emotions.

^b^SSI-GP: single-session intervention growth mindset of personality.

^c^ST: support therapy.

A pilot test among 13 secondary students (ages from 15 to 17 years) was conducted to confirm the feasibility and acceptability of the intervention in a small sample of the targeted population. The intervention lasted around 45 minutes and could be finished in one school class session.

#### SSI-GP

SSI-GP intervention group will use the intervention protocol of project personality [20]. We will translate it into Chinese by 2 bilingual native English and Chinese speakers and make adaptations to the local education context. The key potent elements of SSI-GP consist of five components [15,21,22]: (1) an introduction to the brain about the potential of neuroplasticity and behavioral change; (2) written testimonials from older, high school–aged youths of their belief in change of personality; (3) additional vignettes written by older youths about how growth mindset of personality helped them succeed following setbacks; (4) overview of common questions and misconceptions about growth mindset; and (5) an exercise of writing notes to younger students about the malleability of people’s personality traits.

#### Active Control Group: ST

The control condition will be a structurally similar web-based session of supportive therapy. The goals of supportive therapy are to encourage the client to identify and express feelings and to share their emotions—both positive and negative—with close others. The ST group does not teach or emphasize specific skills or beliefs. The active control group includes the same number of activities as the SIGMA and SSI-GP interventions. Also, to mirror the intervention groups as closely as possible, supportive therapy will include vignettes written by similar school-aged youths, who describe times when they benefited from sharing their feelings with friends or family members.

### Implementation Strategies

As the SSIs in this study are all self-administer web-based interventions, we adopt 5 strategies to ensure the interventions are understandable, relevant, acceptable, engaging, and effective. These strategies will also help promote context sensitivity among school students in the Chinese context.

First, we form a youth advisory group to make sure the intervention content is understandable and relevant to students’ life. The group includes 2 secondary school students of the targeted age group, 2 school social workers, and 2 university students. For example, the testimonials of anxiety situations and coping should be context-adaptive, and the introduction of scientific knowledge should be in plain language and easy to understand. The youth advisory group includes students from the target population, school social workers and counselors, and local university students. We seek their feedback and suggestions during the development and the pilot test of the study to further improve the intervention.

Second, we adopt the philosophy of solution-focused brief therapy. We do not focus on the problem but seek future-oriented solutions relevant to daily living. These techniques can help engage the participants to make the intervention relevant and can ensure the intervention is solution-focused rather than problem-focused.

Third, we integrate “design art and computing science” to make the intervention engaging; for example, we design animation icons and design videos, and use clickers in the self-administrative intervention to maintain the focus of the participants.

Fourth, we empower student participants to act as “helpers” or “experts” by framing the participants as active contributors in the improvement of the intervention and by asking them to provide support to peers [10,11]. This design aligns with adolescents’ desire for respect [22].

Fifth, to ensure the intervention will be conducted in a standardized surrounding, the intervention will be conducted in activity rooms in each school. Around 12 to 20 student participants with parental consent will be invited to participate in the intervention as a group under the support and administration of well-trained research assistants. We also monitor the time spent on each part of the presentation with computing programming techniques. Participants with too long and too short duration time (usually above 2 SDs) in 1 section will be double-checked on their participation effectiveness, and biased data will be eliminated.

### Data Collection and Management

Assessments will be conducted by a blinded researcher trained in using the instruments. Self-rated scales will be completed by the participants under guidance. All the measures have been pilot-tested, and the questionnaires take about 20 minutes to be completed. Two attention-checking items will be included in the surveys of each time point for careless responding screening (eg, “Please choose *Strongly Agree* for this item”). Only those who answer both attention-checking items correctly each time will be included in the final data analysis [23]. As the literature has shown motivation playing pivotal roles in people’s behavioral change, we will also add 2 items at the beginning of the survey at baseline to assess participation motivation of our participants with a 6-point Likert scale, asking about their motivation to participate in this program and improve their emotion regulation ability, respectively.

#### Content Fidelity Checking

##### Mindsets of Negative Emotions

The validated Chinese version 12-item Mindset of Depression, Anxiety and Stress Scale will be used to assess participants’ belief in change of negative emotion states, that is, depression, anxiety, and stress [16]. The sample items are “When you have a certain level of depression, you really cannot do much to change it”; “To be honest, people cannot really change how anxious they are”; and “No matter how hard people try, they cannot really change the level of stress that they have.”

Each item scores on a 6-point Likert scale; a higher score means a more fixed mindset of negative emotions (Cronbach α=.94). There are 3 subscales: depression mindset, anxiety mindset, and stress mindset, containing 4 items in each subscale. The Cronbach α of 3 subscales are .91, .89, and .90, respectively [16].

##### Mindset of Personality

Three items of implicit theory of personality [24,25] will be used to measure the belief in change of personality on a 6-point Likert scale from 1 (*strongly disagree*) to 6 (*strongly agree*). A higher score means a more fixed mindset. The sample item is “People can do things differently, but the important parts of who they are can’t really be changed.” The Cronbach αwas .85.

##### Intervention Feedback Scale

It is developed based on the theoretical framework of acceptability (TFA), which consists of 7 component constructs including affective attitude, burden, intervention coherence, perceived effectiveness, opportunity costs, self-efficacy, and ethicality [26]. Except for a general acceptability item, 6 items for the 6 components of TFA except for ethicality and 4 items including 2 open-ended written feedback items in regards to the impression of the intervention from the well-validated Program Feedback Scale [27] will also be integrated to assess the acceptability of the intervention. Among the final scale, except the open-ended item, the other 10 items will be assessed on a 5-point scale, such as “How acceptable was the intervention to you?” (“1=Completely unacceptable” to “5=Completely unacceptable”).

##### Motivation to Apply Things Learnt From the Program

Right after the intervention, we will ask participants after this program the extent to which they would like to apply the intervention content and the extent to which they would like to improve their emotion regulation on a 6-point Likert scale.

#### Primary Outcome

Anxiety symptoms measured by the Generalized Anxiety Disorder-7 (GAD-7) [28,29] are the primary outcome. The GAD-7 scale includes 7 items that assess whether anxiety symptoms have bothered the individual in the previous 2 weeks, ranging the frequency from 0 (*not at all*) to 3 (*nearly every day*). Example items were “Feeling nervous, anxious, or on edge” and “Not being able to stop or control worrying*.*” GAD-7 is the self-rating scale and effectively reflects symptom severity in adolescents; it is highly correlated with clinician-administered ratings of anxiety symptoms. It is brief and suitable for self-report study [30]. The Cronbach α was .93 [31].

#### Secondary Outcomes

##### Perceived Control

The Anxiety Control Questionnaire-Emotion Control [32] is a 15-item questionnaire that measures how much perceived control participants have over their anxiety. It is 1 of the 3 validated subscales of the anxiety control questionnaire and contains 4 items (eg, *I am able to control my level of anxiety*) rated from 0 to 5. The scale has a well-validated factor structure in a nonclinically selected sample, is strongly associated with anxiety and depression symptoms, and has demonstrated good internal consistency in previous investigations. The Cronbach α was .73.

##### Hopelessness

The 4-item helplessness subscale of the Demoralization Scale [33] will be used to measure the participants’ faith in the future. Each item scores on a 5-point Likert scale, and the mean of all 4 items is taken to measure the hopelessness, with a higher score meaning a correspondingly higher level of hopelessness. An example item is “I feel hopeless.” The Cronbach α of the Chinese version of this helplessness subscale was .72 [34].

##### Attitude Toward Seeking Help

We use 2 items from the Attitude toward Seeking Counselling Help Assessment [32,33], participants’ understanding of counseling and attitudes toward seeking counseling help. An example item is “If I believed I was having a mental breakdown, my first inclination would be to get professional attention*.*” The Cronbach α was .72 [34]. We develop a brief scale including 2 items to assess participants’ help-seeking behavior. The 2 items are “when I encounter difficulties, I will not ask help from teachers” and “when I encounter difficulties, I will not ask help from social workers/counsellors.” This scale will be rated on a 6-point Likert scale from 1 (very untrue of me) to 6 (*very true of me*).

##### Psychological Well-being

The short version Warwick-Edinburgh Mental Well-being Scale (WEMWBS-14) [35,36] will be used to measure the extent to which participants generally experience well-being states. The WEMWBS includes 14 items, scoring on 5-point Likert scale, the average of all 14 items indicates the well-being of the participants. The sample item is “I have been feeling optimistic about the future,” and the Cronbach α was .93.

#### Covariates

Sociodemographic information consists of a range of participant characteristics and is measured at the baseline to examine variability between the groups such as gender, age, grade, ethnicity, and socioeconomic status.

### Data Analysis

An intention-to-treat analysis will be conducted for any missing data. Two-level analyses will be used to account for cluster randomization [37]. To examine the effects of the interventions, multilevel regression will be used to test the group effect, time effect, and their interaction effect on outcome measures. Additionally, we will calculate effect sizes using estimated marginal means; these effect sizes compare mean gain scores (Cohen *d*), reflecting changes in each outcome from baseline to 2 follow-ups for youths receiving the mindset versus active control interventions. The effect sizes will also be compared between participants in the intervention group who receive booster messages and those who do not. A *P* value of <.05 will be considered statistically significant, and SPSS version 26 (IBM Corp) will be used for all statistical analyses.

### Ethics Approval

The study has obtained research ethics approval from the institutional review board of the principal investigator’s university (HSEARS20201004001-01). This trial was preregistered at ClinicalTrials.gov (NCT05027880). Participation will be voluntary. Informed written consent or digital consent will be obtained from parents and participants. We will remind the participants that they have the right to terminate the intervention or omit any questionnaire questions and assure the confidentiality of their answers. We will also provide additional information for referral services to the student participants. Students are also invited to indicate if they need further support from school social workers or counselors in the postintervention questionnaire. With the participant’s assent, we will refer the participant to the school caring professionals. All identifiable information will be removed, and codes will replace names to ensure privacy. All personally identifiable data will be locked up and destroyed 3 years after the study ends. Participants’ data will not be identifiable in any publication or reporting.

## Results

Recruitment will start in December 2022, and data collection is expected to end in 2023. Results are expected to be available in January 2024. We also intend to make the publications available in 2024. The findings of this trial will be presented as well at conferences. We will only report aggregated group data, and no personal information will be leaked.

## Discussion

### Overview

We anticipate that SIGMA, which highlights that emotion states are changeable, will significantly enhance the growth mindset of emotion and reduce general anxiety symptoms and secondary outcomes compared to SSI-GP and the active control. However, the effect size will be small because single-session self-help interventions are low-dosage interventions. SSI-GP is hypothesized to have better outcomes than the active control group. The half with booster messages in the SIGMA group will have better outcomes than the half without boosters, especially in the 8-week follow-up.

To date, this will be one of the few randomized controlled trials to apply a single-session growth mindset intervention to reduce anxiety. As focusing on the key aspects and using effective ways to make changes are the key mechanism of effective SSI, addressing the key aspects that can secure changes is essential for designing SSIs. This 3-arm randomized controlled trial will help elucidate the mechanisms of change in adolescent anxiety symptoms: believing that negative emotion can change (SIGMA), believing that personality can change (SSI-GP), or receiving support (active control). This proposed study will find the potent core element of brief intervention in helping youth with anxiety.

This study will also be a pioneer study examining the self-administered web-based intervention among Chinese adolescents. There is a paucity of research in the Chinese context on effective implementation strategies for self-administrative SSI. As the effect size of SSI varies from small to medium in extant interventions, the dedicated design and careful implementation matter in the efficacy of the study. This study will provide a clear protocol of implementation content and strategies, which will be helpful for the future use and development of SSIs.

This project has theoretical, practical, and clinical contributions. Examining the efficacy of the notion of malleability of negative emotion on youth anxiety will advance the understanding of the impact of implicit theory and mental health symptoms. The easy-access self-help program will enable adolescents with anxiety symptoms to access timely help, and reduce the risk of deterioration of anxiety symptoms and development of comorbid mental health problems before adolescents can access therapy provided by a trained therapist or psychiatrist. If proven effective, SIGMA will benefit a proportion of youths who would otherwise go without service entirely. It can also benefit youth on the waitlist for psychiatric services by invoking intrinsic motivation and avoiding hesitance to get treatment. It can also serve as a complement to multisession psychosocial treatments. This project may provide a generalizable model for the development and implementation of SSI among youth people in the Chinese context.

### Limitations

There are limitations that need to be considered. First, as this study does not exclude individuals based on the severity of their anxiety symptoms, the efficacy of the interventions to reduce anxiety symptoms of students without anxiety or with low levels of anxiety may not be significant and thus affects the statistical significance on the whole. However, the proposed intervention can be inclusive to youths with a diverse range of anxiety problems and offers a unique opportunity to study the differentiated impacts with subgroup analyses. We expect the secondary outcomes will have significant changes after the intervention, even for those with low-level anxiety symptoms. Second, there is no waitlist control group as all 3 groups are implemented with specific interventions, with even the control group receiving ST (active control). In this study, we aim to compare the effects of different interventions with specific strategies. However, the setting of a waitlist control group will help in better understanding the effect of interventions overall.

### Conclusions

This study presents the evidence-based implementation of web-based single-session growth mindset interventions for student anxiety and compares the efficacy of SSIs using growth mindsets on negative emotions and growth mindsets on personality. Our study will provide an example of the implementation of SSIs among the Chinese adolescents and will help to develop easy-access, low-cost, and scalable interventions for mental health promotion among young people.

## Abbreviations

CONSORT: Consolidated Standards of Reporting Trials
GAD-7: Generalized Anxiety Disorder-7
SIGMA: single-session intervention of growth mindset on negative emotions
SSI: single-session intervention
SSI-GP: single-session intervention of growth mindset of personality
ST: support therapy
TFA: theoretical framework of acceptability
WEMWBS: Warwick-Edinburgh Mental Well-being Scale

## Data Availability

Data are not publicly available but can be obtained via contacting the corresponding author.

## References

[1] (2021). Adolescent mental health. World Health Organization.

[2] Leung PWL, Hung SF, Ho TP, Lee CC, Liu WS, Tang CP, Kwong SL (2008). Prevalence of DSM-IV disorders in Chinese adolescents and the effects of an impairment criterion: a pilot community study in Hong Kong. Eur Child Adolesc Psychiatry.

[3] (2018). Survey of depression and anxiety among secondary school students. Baptist Oi Kwan Social Service.

[4] (2020). Student enrolment statistics, 2019/20 (kindergartens, primary and secondary schools). Education Bureau.

[5] Lam LC, Wong CS, Wang MJ, Chan WC, Chen EY, Ng RM, Hung SF, Cheung EF, Sham PC, Chiu HF, Lam M, Chang WC, Lee EH, Chiang TP, Lau JT, van Os J, Lewis G, Bebbington P (2015). Prevalence, psychosocial correlates and service utilization of depressive and anxiety disorders in Hong Kong: the Hong Kong Mental Morbidity Survey (HKMMS). Soc Psychiatry Psychiatr Epidemiol.

[6] (2017). Mental health care services for Hong Kong youth. Bauhinia Foundation Research Centre.

[7] (2020). COVID-19 disrupting mental health services in most countries, WHO survey. World Health Organization.

[8] (2017). Mental health review report. Food and Health Bureau.

[9] Harpaz-Rotem I, Leslie D, Rosenheck RA (2004). Treatment retention among children entering a new episode of mental health care. Psychiatr Serv.

[10] Schleider JL, Dobias ML, Sung JY, Mullarkey MC (2020). Future directions in single-session youth mental health interventions. J Clin Child Adolesc Psychol.

[11] Schleider J, Weisz J (2018). A single-session growth mindset intervention for adolescent anxiety and depression: 9-month outcomes of a randomized trial. J Child Psychol Psychiatry.

[12] Schleider JL, Dobias M, Fassler J, Shroff A, Pati S (2020). Promoting treatment access following pediatric primary care depression screening: randomized trial of web-based, single-session interventions for parents and youths. J Am Acad Child Adolesc Psychiatry.

[13] Schleider JL, Burnette JL, Widman L, Hoyt C, Prinstein MJ (2020). Randomized trial of a single-session growth mind-set intervention for rural adolescents' internalizing and externalizing problems. J Clin Child Adolesc Psychol.

[14] Walton GM (2014). The new science of wise psychological interventions. Curr Dir Psychol Sci.

[15] Walton GM, Wilson TD (2018). Wise interventions: psychological remedies for social and personal problems. Psychol Rev.

[16] Zhu S, Zhuang Y, Lee P (2022). Psychometric properties of the mindsets of depression, anxiety, and stress scale (MDASS) in Chinese young adults and adolescents. Early Interv Psychiatry.

[17] Zhu S, Wong PWC (2022). What matters for adolescent suicidality: depressive symptoms or fixed mindsets? Examination of cross-sectional and longitudinal associations between fixed mindsets and suicidal ideation. Suicide Life Threat Behav.

[18] Schulz KF, Altman DG, Moher D, CONSORT Group (2010). CONSORT 2010 statement: updated guidelines for reporting parallel group randomised trials. BMC Med.

[19] Grant S, Mayo-Wilson E, Montgomery P, Macdonald G, Michie S, Hopewell S, Moher D, on behalf of the CONSORT-SPI Group (2018). CONSORT-SPI 2018 explanation and elaboration: guidance for reporting social and psychological intervention trials. Trials.

[20] Schleider J, Weisz J (2019). Project Personality. OSF Home.

[21] Schleider JL, Weisz JR (2016). Reducing risk for anxiety and depression in adolescents: effects of a single-session intervention teaching that personality can change. Behav Res Ther.

[22] Yeager DS, Dahl RE, Dweck CS (2018). Why interventions to influence adolescent behavior often fail but could succeed. Perspect Psychol Sci.

[23] Oppenheimer DM, Meyvis T, Davidenko N (2009). Instructional manipulation checks: detecting satisficing to increase statistical power. J Exp Soc Psychol.

[24] Chiu CY, Hong YY, Dweck CS (1997). Lay dispositionism and implicit theories of personality. J Pers Soc Psychol.

[25] Zhu S, Zhuang Y, Cheung SH (2020). Domain specificity or generality: assessing the Chinese implicit theories scale of six fundamental psychological attributes. Front Psychol.

[26] Sekhon M, Cartwright M, Francis JJ (2022). Development of a theory-informed questionnaire to assess the acceptability of healthcare interventions. BMC Health Serv Res.

[27] Schleider JL, Dobias M, Sung J, Mumper E, Mullarkey MC (2020). Acceptability and utility of an open-access, online single-session intervention platform for adolescent mental health. JMIR Ment Health.

[28] Spitzer RL, Kroenke K, Williams JBW, Löwe B (2006). A brief measure for assessing generalized anxiety disorder: the GAD-7. Arch Intern Med.

[29] He X, Li CB, Qian J, Cui HS, Wu WY (2010). Reliability and validity of a generalised anxiety disorder scale in general hospital outpatients. Shanghai Arch Psychiatry.

[30] Mossman SA, Luft MJ, Schroeder HK, Varney ST, Fleck DE, Barzman DH, Gilman R, DelBello MP, Strawn JR (2017). The generalized anxiety disorder 7-item scale in adolescents with generalized anxiety disorder: signal detection and validation. Ann Clin Psychiatry.

[31] Zhu S, Zhuang Y, Lee P, Wong PWC (2021). The changes of suicidal ideation status among young people in Hong Kong during COVID-19: a longitudinal survey. J Affect Disord.

[32] Brown TA, White KS, Forsyth JP, Barlow DH (2004). The structure of perceived emotional control: psychometric properties of a revised anxiety control questionnaire. Behav Ther.

[33] Kissane DW, Wein S, Love A, Lee XQ, Kee PL, Clarke DM (2004). The demoralization scale: a report of its development and preliminary validation. J Palliat Care.

[34] Hong XQ (2010). Reliability and validity testing of the Chinese version of the demoralization scale in cancer patients. J Intern Med Taiwan.

[35] Stewart-Brown S, Janmohamed K (2008). Warwick-Edinburgh mental well-being scale (WEMWBS) user guide. Warwick Medical School, University of Warwick.

[36] Ng SSW, Lo AWY, Leung TKS, Chan FSM, Wong ATY, Lam RWT, Tsang DKY (2014). Translation and validation of the Chinese version of the short Warwick-Edinburgh mental well-being scale for patients with mental illness in Hong Kong. East Asian Arch Psychiatry.

[37] Jo B, Asparouhov T, Muthén BO (2008). Intention-to-treat analysis in cluster randomized trials with noncompliance. Stat Med.

